# Dupuytren's contracture and occupational exposure to hand-transmitted vibration

**DOI:** 10.1136/oemed-2013-101981

**Published:** 2014-01-21

**Authors:** Keith T Palmer, Stefania D'Angelo, Holly Syddall, Michael J Griffin, Cyrus Cooper, David Coggon

**Affiliations:** 1 MRC Lifecourse Epidemiology Unit, University of Southampton, Southampton, UK; 2 Institute of Sound and Vibration Research, University of Southampton, Southampton, UK

## Abstract

**Aims:**

The relation between Dupuytren's contracture and occupational exposure to hand-transmitted vibration (HTV) has frequently been debated. We explored associations in a representative national sample of workers with well-characterised exposure to HTV.

**Methods:**

We mailed a questionnaire to 21 201 subjects aged 16–64 years, selected at random from the age-sex registers of 34 general practices in Great Britain and to 993 subjects chosen randomly from military pay records, asking about occupational exposure to 39 sources of HTV and about fixed flexion contracture of the little or ring finger. Analysis was restricted to men at work in the previous week. Estimates were made of average daily vibration dose (*A*(8) root mean squared velocity (rms)) over that week. Associations with Dupuytren's contracture were estimated by Poisson regression, for lifetime exposure to HTV and for exposures in the past week >*A*(8) of 2.8 ms^−2^ rms. Estimates of relative risk (prevalence ratio (PR)) were adjusted for age, smoking status, social class and certain manual activities at work.

**Results:**

In all 4969 eligible male respondents supplied full information on the study variables. These included 72 men with Dupuytren's contracture, 2287 with occupational exposure to HTV and 409 with *A*(8)>2.8 ms^−2^ in the past week. PRs for occupational exposure to HTV were elevated 1.5-fold. For men with an *A*(8)>2.8 ms^−2^ in the past week, the adjusted PR was 2.85 (95% CI 1.37 to 5.97).

**Conclusions:**

Our findings suggest that risk of Dupuytren's contracture is more than doubled in men with high levels of weekly exposure to HTV.

**What is known on this topic**

- The relation between occupational activity and Dupuytren's contracture has long been disputed, but evidence is growing that exposure to hand-transmitted vibration (HTV) is a risk factor for the disease.

**What this study adds**

- In a large national sample of workers with well-characterised exposure to HTV we found that risks of fixed flexion deformity affecting the ring or little finger were increased almost threefold in workers with a relatively high estimated level of exposure in the previous week.
- Risks were also increased in relation to certain manual activities in a typical working week (heavy lifting, digging and shovelling).
- Associations between HTV and Dupuytren's contracture persisted after adjustment for age, social class and manual occupational activities.

## Introduction

In 1831, when describing the disorder that now bears his name, Baron Guillaume Dupuytren noted an association with hand activities in manual trades.^1^ The relation between occupation and Dupuytren's contracture has subsequently been disputed,^2–4^ but evidence on risks from hand-transmitted vibration (HTV) and heavy manual work has grown and a recent meta-analysis of studies published between 1951 and 2007, by Descatha *et al*, estimated a more than doubling of risk from these exposures.^5^

The threshold of a doubling of risk has been adopted by the UK's Industrial Injuries Advisory Council as a cut-off point in recommending entitlement to state compensation for occupationally related illness (based on the logic that, if the association with exposure is causal, the attributable fraction in exposed claimants will exceed 50% and the disease will be attributable to work on the balance of probabilities^6^). However, especially in relation to HTV, Descatha *et al* identified comparatively few reports to inform appraisal of risks. To inform the Council's deliberations on Dupuytren's contracture and HTV, we revisited data from a national survey of vibration undertaken in Great Britain in 1997–1998 and analysed risks of the disease in relation to a well-characterised assessment of occupational exposures to HTV in a nationally representative sample.

## Methods

Full details of the sampling and methods of this cross-sectional survey are published elsewhere.^7^ ^8^ In brief, the study sample comprised 21 422 men and women aged 16–64 years, selected at random from the patient lists of 34 general practices, and 993 members of the armed services chosen randomly from central pay registers, but 221 patients were excluded on general practitioners’ advice. The practices were chosen to give a broad geographical coverage of Great Britain, while members of the armed forces (who have different provisions for primary care) were included in a separate mailing and selected at random from central military pay records. The questionnaire, which was developed in consultation with various health and safety professionals, vibration specialists, trades unions and trade associations,^9^ included, among other things, questions on current occupation and industry; social class (manual, non-manual, armed services); exposures to HTV at work in the previous 7 days (sources and durations of exposure); exposures to HTV in previous paid jobs for >1 h/week; age, sex and smoking habits; certain physical activities in a typical work day (lifting weights >56 pounds (roughly 25 kg), digging or shovelling, use of a computer keyboard for >4 h/day); and Dupuytren's contracture.

Information on current exposure to HTV was obtained principally from a question about use in the past week of 39 listed tools and machines for which dominant axis frequency-weighted vibration accelerations (*a*_hw_ values) could be assigned from published measurements and other information held by the Institute of Sound and Vibration Research^10^; from these and usage times, average personal daily vibration exposures (*A*(8)) for the past week were estimated assuming the time dependency in ISO 5349–1, 2001 and summing the partial doses arising from each source, as described elsewhere.^7^ ^8^ At the time of the survey, the UK's Health and Safety Executive had suggested that when exposures exceeded the equivalent of 2.8 ms^−2^ for 8 h (*A*(8) > 2.8 ms^−2^ root mean squared velocity (rms)), control measures and a programme of health surveillance would be required (a threshold since lowered^11^ to 2.5 ms^−2^), and the present analysis uses 2.8 ms^−2^ as a cut-off point in defining higher levels of exposure. Open questions on exposure to HTV in previous paid jobs were also asked, and these were coded by a panel of vibration specialists to classify subjects’ lifetime exposure (ever vs never) to relevant occupational tools.

**Figure 1:**
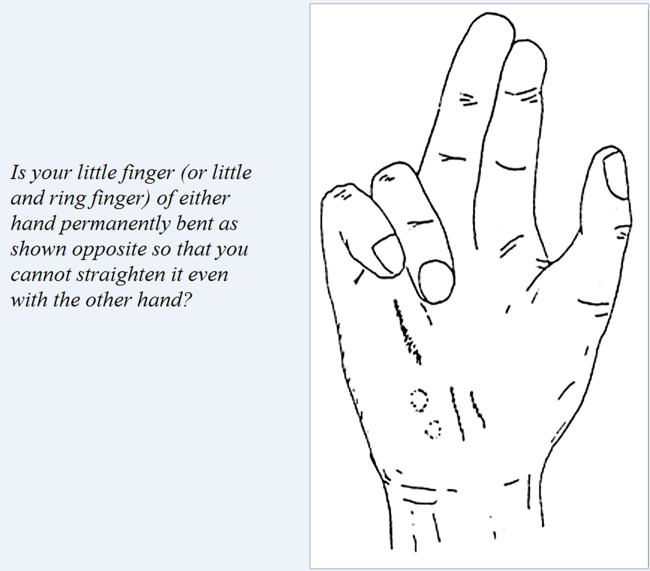
The question used to ascertain Dupuytren's contracture.

Dupuytren's contracture was assessed by means of a single question: “Is your little finger (or little and ring finger) of either hand permanently bent as shown opposite so that you cannot straighten it, even with the other hand?” The question was accompanied by a line drawing (figure 1).

As exposure to HTV was far more common in men than women, we restricted analysis to male respondents with non-missing information on the above variables, and who held a paid job and were at work in the week before the questionnaire was completed. Associations with Dupuytren's contracture were assessed by Poisson regression (using robust SEs), according to any relevant occupational exposure to HTV and according to whether exposures to HTV had occurred in the past week or not, and were above or below HSE's action threshold (2.8 ms^−2^). Models were adjusted initially for age in three bands (16–44 years, 45–54 years and 55–65 years) and then also for the other variables—that is, smoking status (ever vs never), social class, lifting weights >56 lbs, digging/shovelling and use of a computer keyboard for >4 h/day. (The latter was included to test the specificity of association with physical activity and as a marker of potential reporting bias—specifically, the propensity to over-report work hand activities in general when limited by fixed flexion deformity of a digit.) Findings were expressed as prevalence ratios (PRs) with associated 95% CIs (95% CI). All analyses were performed in Stata (V.13).

## Results

In all, 22 194 subjects were mailed a questionnaire and 12 907 (58% or 61% of those with valid postal addresses) returned usable responses. These included 6913 men; but 1423 of the male respondents were unemployed, 126 had been absent from work in the previous week, and a further 395 failed to provide complete information on the variables to be analysed (with missing data on vibration exposure (N=204), social class (132), Dupuytren's contracture (55) and age (4)). Thus, the final sample comprised 4969 employed or self-employed men (95% of respondents in work over the relevant period).

Dupuytren's contracture was reported by 72 (1.4%) of these men. In all, 2287 men (46% of the sample) reported occupational exposure to one or more of the vibratory tools, including 409 men (8.2%) whose *A*(8) in the past week was estimated to exceed 2.8 ms^−2^.

Table 1 presents the prevalence of Dupuytren's contracture according to age, smoking status, social class and physical activities in a typical working day; also, estimates of PRs for these factors, with adjustment for age. The prevalence of and PR for Dupuytren's contracture rose sharply with age, and the outcome was significantly more common in manual workers and men undertaking heavy lifting, and digging or shovelling at work; but no significant association was found with smoking or with use of a computer keyboard.

**Table 1:** Prevalence of Dupuytren's contracture and its relation to demographic and occupational activities

Characteristic		Dupuytren's contracture present		
	N	N	(%)	PR (95% CI)
Age (years):				
16–44	3066	30	0.98	1
45–54	1273	19	1.49	1.53 (0.86 to 2.70)
55–65	630	23	3.65	3.73 (2.18 to 6.38)
Smoking:				
Never	2380	28	1.18	1
Ever	2589	44	1.70	1.22 (0.76 to 1.97)
Social class:				
Non-manual (I,II, IIINM)	2161	23	1.06	1
Manual (IIIM, IV, V)	2303	44	1.91	1.76 (1.06 to 2.90)
Armed forces	505	5	0.99	1.36 (0.52 to 3.59)
Lifting weights*:				
No	2508	30	1.20	1
>20 lbs	2461	42	1.71	1.48 (0.94 to 2.35)
>56 lbs	1627	31	1.91	1.64 (1.04 to 2.60)
Digging/shovelling*:				
No	4333	57	1.32	1
Yes	636	15	2.36	1.87 (1.07 to 3.28)
Use of keyboard (>4 h)*:				
No	3847	60	1.56	1
Yes	1122	12	1.07	0.79 (0.42 to 1.46)

Each risk factor was assessed in a separate model; all models were adjusted for age (in three bands).

*In an average working day.

Table 2 explores associations with HTV, overall and then for several mutually exclusive categories of exposure, with risk estimates adjusted for age (model 1), then for age and social class (model 2) and then for all of the variables in table 1 (model 3). With simple adjustment for age, the PR for any occupational exposure to HTV (ever vs never) was 1.87. PRs were also significantly elevated for exposure to HTV in the previous week, more so when the *A*(8) was >2.8ms^−2^ (PR 3.48) than when it was ≤2.8 ms^−2^ (PR 1.68). In the fully adjusted model, risk was elevated about 1.5-fold for a history of ever being occupationally exposed to HTV, while the PR for exposure in the past week above 2.8 ms^−2^ was 2.85 (95% CI 1.37 to 5.97).

**Table 2:** Relation between Dupuytren's contracture and occupational exposure to hand-transmitted vibration

Exposure to hand-transmitted vibration		Dupuytren's		Model 1*	Model 2*	Model 3*
	N	N	(%)	PR (95% CI)	PR (95% CI)	PR (95% CI)
Never	2682	28	1.04	1	1	1
Ever	2287	44	1.92	1.87 (1.17 to 2.98)	1.65 (1.00 to 2.67)	1.53 (0.93 to 2.51)
Ever, but not in the past week	710	9	1.27	1.28 (0.61 to 2.70)	1.25 (0.59 to 2.65)	1.23 (0.58 to 2.62)
Past week, *A*(8)<=2.8 ms^−2^	1168	21	1.80	1.68 (0.96 to 2.94)	1.53 (0.86 to 2.72)	1.51 (0.82 to 2.79)
Past week, *A*(8)>2.8 ms^−2^	409	14	3.42	3.48 (1.85 to 6.53)	3.06 (1.46 to 6.40)	2.85 (1.37 to 5.97)

*Model 1: adjusted for age (in three bands); Model 2 adjusted for age and social class; Model 3: adjusted for age and the other factors listed in table 1.

## Discussion

This analysis, based on a large population survey of occupational exposure to vibration and health, suggests that HTV increases the risk of Dupuytren's contracture, and that PRs can be elevated, some threefold, with higher exposures.

Our dataset has some notable strengths, but also certain limitations. The sample originally selected for study was chosen to provide information that within each occupation would be representative of Britain as a whole; it included more than 21 000 adults from general practices across the country (almost everyone registers with a National Health Service general practitioner, apart from members of the armed forces who were sampled separately). Quantitative estimates of exposure to HTV are subject to several potential sources of error and bias,^7^ ^12^ but estimates from this study were particularly well characterised. They were based principally around a predefined checklist of sources (identified by literature review, by CEN/TC 231/WG2 as requiring type testing, and by a broad panel of vibration specialists and stakeholders),^9^ together with independent workplace visits to confirm the accuracy of exposure reporting^12^ and independent field measurements of exposure for sources that were common in the survey.^10^

Set against this, the survey focused principally on exposures over the previous week and did not seek to quantify lifetime cumulative exposure to HTV—a task that is fraught with potential for error. Thus, the exposures analysed here are only a proxy for those of greatest interest. Misclassification of exposure in these circumstances is liable to be non-differential, however, and should, if anything, lead to an underestimation of risks.

Another limitation of our study is that we were not able to examine respondents clinically, to corroborate the diagnosis of Dupuytren's contracture. We believe that the question and line drawing in figure 1 have acceptable face validity, but this has not been tested empirically; nor would our question identify the milder early stages of disease in which nodules and palmar thickenings occur without fixed flexion contracture. However, it seems unlikely that deficiencies in questioning could explain the gradient found with estimated exposure to HTV; nor is it likely to have arisen from response bias, since this particular inquiry would have been relatively disguised from respondents (a single item in a 20-page questionnaire on exposures to vibration), and the ‘dummy’ hand activity we analysed (use of a computer keyboard) showed no association with flexion contracture.

We restricted analysis to a cross section of men at work in the previous week. Risks could have been underestimated if Dupuytren's contracture caused men to quit exposed jobs for unexposed ones or to leave employment altogether (unhealthy worker selection bias). Only the latter would bias risk estimates in relation to lifetime exposure to HTV, but the former could have reduced the apparent exposure-risk gradient to the extent that this comparatively mild disease prompts job change. In theory, bias might also have arisen to the extent that Dupuytren's contracture is surgically correctable and (in the absence of selection out of work) manual workers accessed surgery less (or more) readily than non-manual workers. We have no information on subjects’ past surgical history, but judge the likely impact to be small, since most cases of the disease cause minor disability and are not so treated.

Statistical power in our study was limited by the age of those studied (disease prevalence is comparatively low at working ages and rises substantially in later life), but we were still able to detect PRs that were significantly elevated at the 5% level.

Dupuytren's disease has a number of established non-occupational risk factors beyond age. These include genetic constitution (eg, the disease is commoner more common in Nordic populations), smoking, heavy alcohol consumption, epilepsy, anticonvulsants and diabetes mellitus.^4^ Among these and other potential confounders, we were able to adjust only for age and smoking, as data on the other variables were not collected. It seems unlikely, however, that the observed dose-related effects of HTV could arise from confounding by comparatively uncommon risk factors in the population at large. The disease may also arise from the physical labour of work,^1^ ^5^ and adjustment for manual social class and certain physical aspects of respondents’ jobs reduced estimated PRs for exposure above 2.8 ms^−2^ in the past week by some 20%. However, they remained substantially and significantly elevated.

In keeping with our study, relative risks of 2 to 3 (with evidence of a dose-response relationship) were reported in a cross-sectional study of Italian quarry drillers and stone carvers with an average of 17.4 years of exposure to relatively high levels of HTV^13^; by about fivefold to 11-fold in manual workers employed by private companies in the Pays de Loire region of France^14^; by almost twofold in male users of powered tools from a different French survey who had been exposed for a median of 10 years^15^; by twofold to threefold in Italian men from a broad range of occupations (mine driller, stone cutter, stone dresser, building worker, chainsaw user, timber worker, milling worker, grinder, polisher) when exposed for ≥10 years^16^; and, also, by almost twofold in men claiming vibration-induced white finger when compared with similarly aged men from a general surgical ward of a hospital in England.^17^ By contrast, a very large British study, involving over 97 000 miners and ex-miners seeking compensation for Hand-arm Vibration Syndrome, found no relationship with years of exposure to HTV when analysed as a continuous variable.^18^ However, the extent of exposure contrast within this selected group of heavily exposed claimants is unclear and no analysis was provided by exposure in categorical bands. (The chosen approach assumes that exposure increases disease risk exponentially, possibly overlooking an alternative shape of exposure-response^1^,^9^)

In Descatha's review^5^ (which excluded this last study of miners because it lacked a control group), the meta-relative risk for vibration at work was 2.88 (95% CI 1.36 to 6.07), and 2.14 (95% CI 1.59 to 2.88) in a sub-analysis confined to reports of higher quality. Our findings from the present survey, especially the fully adjusted risk estimates for exposures >2.8 ms^−2^, are remarkably close to Descatha's meta-estimate of effect. We judge the balance of evidence now to support a rough doubling in such risks assuming a decade or more of exposure to HTV at substantial levels of vibration.

## References

[1] Dupuytren G Permanent retraction of the fingers produced by an affection of the palmar fascia. Lancet 1834;2:222–5

[2] McFarlane RM Dupuytren's disease: relation to work and injury. J Hand Surg (Am) 1991;16:775–9 1834729 10.1016/s0363-5023(10)80134-0

[3] Burge CP Dupuytren's disease. J Bone Joint Surg (Br) 2004;86B:1088–90 15446545 10.1302/0301-620x.86b7.15766

[4] Townley WA Baker R Sheppard N Dupuytren's contracture unfolded. BMJ 2006;332:397–400 16484265 10.1136/bmj.332.7538.397 PMC1370973

[5] Descatha A Jauffret P Chastang J-F Should we consider Dupuytren's contracture as work-related? A review and meta-analysis of an old debate. BMC Musc Dis 2011;12:96 10.1186/1471-2474-12-96 PMC3123614 21575231

[6] Harrington JM Newman Taylor AJ Coggon D Industrial injuries compensation. Br J Ind Med 1991;48:577–8 1832938 10.1136/oem.48.9.577 PMC1035425

[7] Palmer KT Griffin MJ Bendall H Prevalence and pattern of occupational exposure to hand-transmitted vibration in Great Britain: findings from a national survey. Occup Environ Med 2000;57:218–28 10810107 10.1136/oem.57.4.218 PMC1739937

[8] Palmer KT Coggon DN Bendall HE Hand-transmitted vibration: Occupational exposures and their health effects in Great Britain. HSE Contract Research Report 232/1999, Sudbury, HSE Books, 1999

[9] Palmer K Coggon D Griffin M The development of a self-administered questionnaire to assess exposures to hand-transmitted and whole body vibration and their health effects. J Sound Vib 1998;215:653–86

[10] Paddan GS Haward BM Griffin MJ Hand-transmitted vibration: Evaluation of some common occupational sources of exposure in Great Britain. HSE Contract Research Report 234/1999, Sudbury, HSE Books, 1999

[11] Health and Safety Executive Health surveillance for Hand-arm vibration syndrome. http://www.hse.gov.uk/vibration/hav/advicetoemployers/havsemployers.pdf (accessed 19 Aug 2013).

[12] Palmer KT Haward B Griffin MJ Validity of self-reported occupational exposures to hand-transmitted and whole-body vibration. Occup Environ Med 2000;57:237–41 10810109 10.1136/oem.57.4.237 PMC1739940

[13] Bovenzi M Hand-arm vibration syndrome and dose-response relation for vibration induced white finger among quarry drillers and stonecarvers. Occup Environ Med 1994;51:603–6 7951792 10.1136/oem.51.9.603 PMC1128054

[14] Descatha A Bodin J Ha C Heavy manual work, exposure to vibration and Dupuytren's disease? Results of a surveillance program for musculoskeletal disorders. Occup Environ Med 2012;69:296–2 22213840 10.1136/oemed-2011-100319 PMC3815440

[15] Lucas G Brichet A Roquelaure Y Dupuytren's disease: Personal factors and occupational exposure. Am J Ind Med 2008;51:9–15 18033693 10.1002/ajim.20542

[16] Cocco Frau P Rapallo M Casula D Occupational exposure to vibration and Dupuytren's disease: a case-controlled study. Med Lav 1987;78:386–92 3447028

[17] Thomas PR Clarke D Vibration white finger and Dupuytren's contracture: are they related? Occup Med 1992;42:155–8 10.1093/occmed/42.3.155 1345125

[18] Burke FD Proud G Lawson IJ An assessment of the effects of exposure to vibration, smoking, alcohol and diabetes on the prevalence of Dupuytren's disease in 97,537 miners. J Hand Surg Eur Vol 2007; 32:400–6 17950195 10.1016/J.JHSE.2005.02.002

[19] Checkoway H Pearce N Kriebel D Research methods in occupational epidemiology. 2nd ed. New York: Oxford University Press, 2004:280–2

